# Roles of circadian clocks in cancer pathogenesis and treatment

**DOI:** 10.1038/s12276-021-00681-0

**Published:** 2021-10-07

**Authors:** Yool Lee

**Affiliations:** Department of Translational Medicine and Physiology, Elson S. Floyd College of Medicine, Washington State University, Spokane, WA 99202 USA

**Keywords:** Circadian rhythms, Targeted therapies

## Abstract

Circadian clocks are ubiquitous timing mechanisms that generate approximately 24-h rhythms in cellular and bodily functions across nearly all living species. These internal clock systems enable living organisms to anticipate and respond to daily changes in their environment in a timely manner, optimizing temporal physiology and behaviors. Dysregulation of circadian rhythms by genetic and environmental risk factors increases susceptibility to multiple diseases, particularly cancers. A growing number of studies have revealed dynamic crosstalk between circadian clocks and cancer pathways, providing mechanistic insights into the therapeutic utility of circadian rhythms in cancer treatment. This review will discuss the roles of circadian rhythms in cancer pathogenesis, highlighting the recent advances in chronotherapeutic approaches for improved cancer treatment.

## Cancer: Reviewing the role of circadian rhythm

Therapies that reset the body’s circadian clock could prove valuable in treating multiple cancers. Chronic disruption to circadian rhythm by genetic and environmental factors such as frequent jet lag is associated with increased susceptibility to diseases, particularly cancer. Yool Lee at Washington State University in Spokane, USA, reviewed understanding of clock dysregulation in cancers and the promising application of novel ‘chronotherapies’. Disruption to the molecular crosstalk between clock mechanisms and cell cycles (including cell death and immune pathways) can allow cancer cells to take hold. Rhythm disruption also modulates the activity of tumor suppressors and cancer-promoting genes, and can even trigger anti-cancer drug resistance. Training the clock via light therapy, exercise, and administering chemotherapy drugs at specific times of day may prove valuable, while drugs that affect the circadian clock also show promise.

## Introduction

Circadian clocks are cell-autonomous timing systems that generate approximately 24-h periodic rhythms that are conserved in nearly all life, from unicellular organisms to humans. These internal timing systems integrate diverse environmental (e.g., light) and metabolic (e.g., food intake) stimuli to regulate biological activities, including sleep/wake, energy metabolism, hormonal and immune functions, and cell proliferation^1^. Disturbances in these rhythms caused by sleep deprivation, eating at night, or chronic jet lag are closely associated with the development of sleep and mood disorders, obesity, diabetes, and cancers^2^. In particular, genetic and environmental perturbations of circadian rhythms largely alter the expression and activity of several tumor suppressors and oncogenes in both host and tumor tissues to favor cancer incidence and progression^3,4^. Circadian disruptions can also reprogram host metabolism and immune systems, fostering an immunosuppressive tumor microenvironment in multiple cancer types^5,6^. Due to such roles in cancer onset and progression, circadian rhythms have been an emerging target for cancer prevention and treatment. In recent years, growing numbers of studies have investigated the idea of exploiting circadian clocks for cancer therapy by enhancing circadian rhythms, modulating the activity of circadian clock molecules, and optimizing the timing of anticancer drugs according to the host or tumor circadian rhythms^7^. In this review, we will discuss additional details regarding the link between circadian clocks and cancer pathways, highlighting treatment strategies that exploit multiple chronophysiological mechanisms.

### Mechanism of circadian physiology

**Fig. 1:**
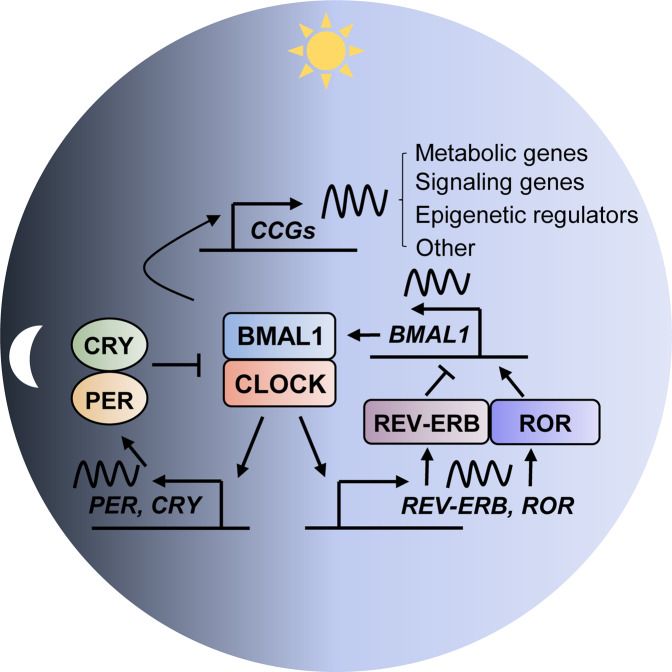
Circadian molecular clock mechanism. This autoregulatory feedback loop cycles between the CLOCK/BMAL1 transcriptional activator complex and its repressors (PER/CRY, REV-ERBα) or activators (RORα/β) to constitute the molecular clock oscillator that drives the expression of multiple clock-controlled genes (CCGs), such as metabolic genes, signaling genes, and epigenetic regulators.

The basic architecture of circadian rhythm mechanisms across living species on earth is typically characterized by a cell-autonomous autoregulatory feedback loop^8^. In eukaryotes, a subset of dedicated positive and negative clock regulators forms the interlocked transcriptional translational feedback loop to constitute a cell-autonomous oscillator that drives the rhythmic expression of output genes involved in metabolic, biosynthetic, signal transduction, and cell cycle pathways^8,9^. In mammals, the BMAL1 and CLOCK transcriptional activator complex cyclically drive the transcription of its own repressors, period (PER), and cryptochrome (CRY). The core oscillator is complemented by a second loop in which periodic expression of BMAL1 is maintained by the REV-ERBα/β repressor and retinoic acid receptor-related orphan receptor (ROR) α/β activator proteins^10^ (Fig. 1). In addition to the core regulatory loops, multiple levels of epigenetic, posttranscriptional, and posttranslational regulation that involve various kinases and phosphatases, ubiquitin–proteasome pathway components, nuclear–cytoplasmic transporters, noncoding RNAs, and chromatin remodelers contribute to the molecular clockwork, thus coordinating temporal programs via multiple clock-output genes^8,11,12^. This molecular clockwork is shared across the brain and peripheral organ systems, constituting a body-wide circadian network.

**Fig. 2:**
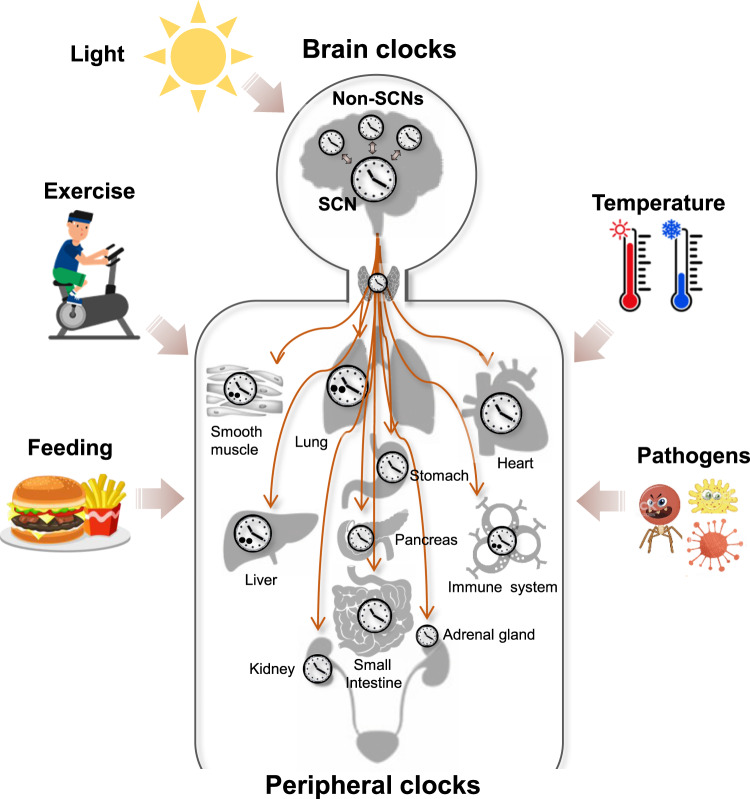
Circadian clock systems. The SCN central clock in the brain, primarily entrained by light, orchestrates circadian phases not only in non-SCN subordinate brain clocks via rhythmic release of neurotransmitters and neuropeptides but also in peripheral organ clocks via systemic hormonal secretion and neural innervations. Nonphotic external cues (e.g., temperature changes, food intake, exercise, and pathogens) can reset circadian rhythms in peripheral clock tissues, thereby influencing rhythmic output physiology and behaviors.

In the brain, intracellular oscillators in approximately 20,000 individual neurons and astrocytes comprise the hypothalamic suprachiasmatic nucleus (SCN), a central circadian pacemaker^13^. The principal role of the SCN clock is to communicate retinal light information received from the retinohypothalamic tract (RHT) to peripheral clock systems, thus mediating the periodic synchronization of internal body rhythms with external day and night cycles^1^. In a hierarchical organization model, the SCN master clock orchestrates circadian phases in non-SCN subordinate brain clocks via rhythmic release of neurotransmitters and neuropeptides, as well as in peripheral organ clocks via systemic hormonal secretion and neural innervations, thereby coordinating rhythmic output physiology and behaviors with daily environmental changes^14^ (Fig. 2). For example, the SCN coordinates the rhythmic, antiphasic secretion of the night sleep hormone melatonin from the pineal gland with the release of the morning stress hormone GC from the adrenal glands via the sympathetic nervous system to ensure daily rhythms in sleep/wake, as well as neural, metabolic, and immune functions^1^. In addition, the brain master clock controls other peripheral clock functions in the heart, kidney, pancreas, lung, intestine, and thyroid glands through circadian modulation of the autonomic nervous system^14^.

In addition to SCN-dependent clock entrainment, non-SCN brain regions and peripheral tissues possess their own autonomous and multistimuli entrainable oscillators that influence not only circadian functions in the SCN and neighboring local clocks but also behavioral rhythms via neural, hormonal, and metabolic feedback mechanisms^15,16^. Moreover, multiple nonphotic physiological (e.g., redox cofactors, reactive oxygen species, microbial products) and environmental (e.g., temperature, food intake, exercise, and pathogenic infections) cues control extra-SCN brain and peripheral clock functions, which, in turn, impact the entire host clock system via neural and immunometabolic circuits^17–19^. These findings suggest that real-world circadian rhythms are achieved through multimode regulation of tightly coupled body clocks under daily changes in internal and external processes.

### Circadian disruption and cancer pathogenesis

**Fig. 3:**
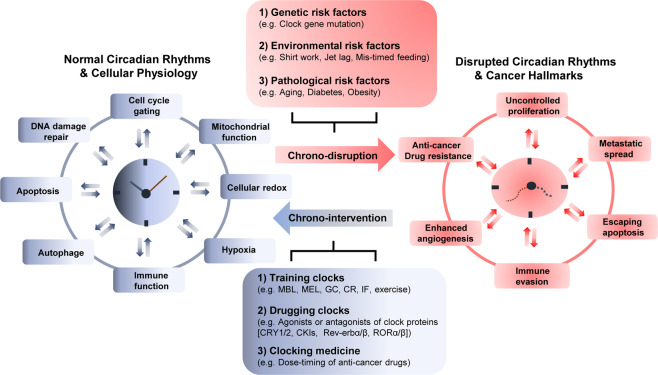
Chronodisruptive factors and chronotherapeutic interventions in cancer pathogenesis and treatment. Circadian clocks reciprocally interact with multiple pathways involved in cellular homeostasis and metabolism. Circadian rhythm disruptions caused by genetic, environmental, and pathological risk factors promote cancer onset and progression characterized by cancer hallmarks. Conversely, several types of chronotherapeutic interventions can enhance or restore circadian rhythms to reduce cancer pathogenesis and improve the response to anticancer treatment. MBL morning bright light, MEL melatonin, GCs glucocorticoids, CR caloric restriction, IF intermittent fasting.

The growing knowledge of chronobiological mechanisms has increased our understanding of how rhythm disturbances by multiple physiological and environmental factors impact pathogenesis in diseases such as cancer (Fig. 3). Disrupted circadian rhythmicity has been a prominent feature of modern society since the invention of artificial light sources. Indeed, 80% of the world’s population is now exposed to light during the night, and approximately 18–20% of workers in the USA and Europe are involved in night or rotating shift work, making them vulnerable to multiple rhythm disorders, including cancer^20^. Several epidemiological studies further suggest that night shift work or chronic jet lag increases the risk of the incidence and development of the most common cancer types (i.e., breast, lung, prostate, colorectal, and skin cancers)^2^. Based on this evidence, the International Agency for Research on Cancer (IARC) classified “shift-work that involves circadian disruption” as potentially “carcinogenic to humans (Group 2A)” in 2007 and again in 2019^21^.

The results from several studies of animal models exposed to forced circadian desynchrony regimens have also reinforced the causal relationship between circadian disturbances and cancer pathogenesis. In an earlier mouse model study, SCN ablation or exposure to experimental chronic jetlag (CJL, consisting of an 8-h advance of the light-dark cycle every 2 days) was shown to cause alterations in circadian physiology and significantly accelerate the growth rates of transplanted tumors (Glasgow osteosarcoma and pancreatic adenocarcinoma)^22^. Subsequent animal studies showed that circadian rhythm disruptions induced by CJL promoted lung tumor growth and progression with altered immune functions^4,23^. Furthermore, CJL conditions have been observed to accelerate tumor cell cycle progression and growth rates in carcinogen-induced tumors as well as grafted melanomas in mice^3^. In line with these results, another murine melanoma model study reported that circadian disruption facilitates tumor-immune microenvironment remodeling that favors tumor cell proliferation^6^. More recently, chronic circadian disruption has also been shown to promote breast cancer cell dissemination and metastasis in mice by increasing the stemness and tumor-initiating potential of tumor cells and by creating an immunosuppressive shift in the tumor microenvironment^5^.

### Roles of circadian clock components in cancer

**Fig. 4:**
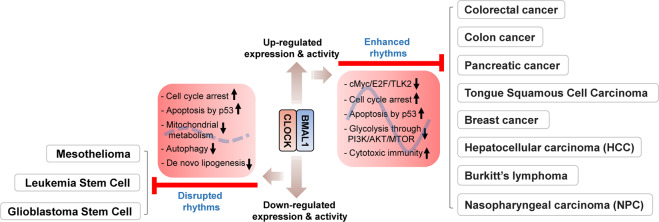
Divergent roles of circadian clock components in cancer. Enhanced levels or activity of circadian clock components (e.g., CLOCK/BMAL1) inhibit tumor proliferation and growth by promoting the degradation of oncoproteins (e.g., cMYC, E2F, and TLK2), cell cycle arrest, apoptotic cell death, metabolic defects, and cytotoxic immunity in multiple cancers, as indicated. On the other hand, the core clock components may also exert tumor-suppressive functions in some cancer cell types (e.g., mesothelioma, leukemia stem cells, glioblastoma stem cells) by inhibiting tumor progression upon downregulation. E2F early 2 factor, TLK2 tousled like kinase 2.

At the molecular and cellular levels, there is close crosstalk between the circadian clock machinery and the cell cycle, DNA repair, apoptosis, senescence, autophagy, and other oncogenic and immune pathways^7^. Circadian perturbations dysregulate these processes, leading to uncontrolled proliferation, escape from apoptosis, metastatic spread, immune evasion, enhanced angiogenesis, and anticancer drug resistance, which are all hallmarks of cancer^24^ (Fig. 3). In this regard, multiple loss- and gain-of-function studies with cellular and animal models have demonstrated the direct involvement of clock genes in cancer predisposition and development. Epigenetic or genetic inactivation of *Bmal1* and/or *Clock* has been shown to increase tumor proliferation or growth rates in several types of cancer, such as hematologic cancer^25^, colon cancer^26^, pancreatic cancer^27^, tongue squamous cell carcinoma (TSCC)^28^, breast cancer^29^, lung adenocarcinoma^4^, hepatocellular carcinoma (HCC)^30^, nasopharyngeal carcinoma (NPC)^31^, and glioblastoma (GBM)^32^. Conversely, overexpression of these circadian activators suppresses proliferative and malignant phenotypes in tumor cells via mechanisms involving cell cycle arrest and p53-dependent apoptosis^32,33^. Similarly, downregulation or upregulation of *Per1*, *Per2*, and *Cry1*, the principal target genes of BMAL1/CLOCK, has been shown to promote or suppress tumor incidence and proliferation, respectively, in multiple cancer cell types, including Lewis lung carcinoma and mammary carcinoma cells^34^, pancreatic cancer^35^, lung carcinoma^4^, leukemia^36,37^, glioma^38^, ovarian cancer^39^, and oral squamous cell carcinoma^40,41^. The potential anticancer mechanism exerted by PER1/2 has been suggested to involve the inhibition of PI3K/AKT/mTOR-mediated glycolysis as well as cell cycle arrest and apoptosis induction^34,40,41^. As an additional tumor-controlling mechanism, the Lamia group has reported that CRY1 and/or CRY2 promote the degradation of cMYC, early 2 factors (E2F) family members, and tousled-like kinase 2 (TLK2) by recruiting these cell cycle-related oncogenic factors to the SCF^FBXL3^ ubiquitin-ligase complex^42^. Consistent with these results, a recent large-scale systems analysis of 32 human cancer types revealed that PERs and CRYs, among several other clock genes, are downregulated in multiple cancers^43^. Overall, these findings highlight the tumor suppressor functions of canonical clock components in most cancer types (Fig. 4).

On the other hand, some studies show that the core clock genes exert tumor promotive functions, depending on the cancer cell status or type. For example, BMAL1 silencing was observed to lead to a substantial increase in apoptosis with mitotic and morphological abnormalities in malignant pleural mesothelioma (MPM) cells^44^. Similarly, knockdown of either *BMAL1* or *CLOCK* has been observed to induce cell cycle arrest and apoptosis in cancer stem cells (CSCs) in patient-derived GBM^45^ or murine leukemia stem cells (LSCs) in acute myeloid leukemia (AML)^46^. Furthermore, a recent study showed that BMAL1 overexpression promotes breast cancer cell invasion and metastasis by upregulating the expression of matrix metalloproteinase 9 (MMP9), a mediating factor for local invasion and distant metastasis of tumors^47^. More recently, CRY1 has been suggested to be a critical factor for efficient DNA repair in tumors, acting as a protumorigenic factor^48^. This is reminiscent of previous studies showing that knockout of *Cry1/2*^*−/−*^ in mice enhances apoptosis pathways in genotoxic responses to UV or cisplatin, a DNA damaging agent, by causing increased expression of the proapoptotic Factor p73^49^. The tumor-promoting function of CRY1 has been further suggested in a recent study showing that CRY1 enhances p53 tumor suppressor degradation via p53 binding to its ubiquitin E3 ligase MDM2 proto‑oncogene in bladder cancer cells, thereby increasing anticancer drug sensitivity^50^. Altogether, these results suggest divergent roles of circadian clock genes in tumor pathogenesis depending on the type and/or state of the cell (Fig. 4).

### Targeting circadian rhythms in cancer treatment

Further insights into circadian rhythms and their related diseases have ignited growing interest in how these processes can be utilized to improve cancer prevention and treatment. In a recent review article, Sulli et al. nicely categorized chronotherapeutic approaches into three types: (1) training the clock: interventions to enhance or maintain a robust circadian rhythm in feeding-fasting, sleep-wake, or light–dark cycles; (2) drugging the clock: using small-molecule agents that directly target a circadian clock; and (3) clocking the drugs: optimizing the timing of drugs to improve efficacy and reduce adverse side effects^51^ (Fig. 3). These chronobiological concepts are not exclusive to each other but have been applied to cancer treatment in a combinatory manner. For example, training the clock can be implemented to strengthen the compromised or disordered rhythms, thus improving the effects of clocked drugs or drugging the clock. With these strategies in mind, in the following sections, we describe recent chronobiological approaches in cancer therapy.

#### Training circadian clocks for cancer treatment

Based on the rhythm entrainment mechanism by photic stimuli, morning bright light (MBL) exposure has been widely implemented to cure sleep problems (e.g., advanced or delayed sleep phase syndromes), neuropsychiatric diseases (e.g., autism spectrum disorder, attention deficit hyperactivity disorder, and dementia), and metabolic disorders (e.g., diabetes and obesity) in preclinical or clinical settings^52^. In line with this, light therapy has been an emerging chronotherapeutic tool to mitigate cancer progression. Accumulated studies with a human cancer xenograft model have revealed that daytime blue light enhances nighttime circadian melatonin inhibition of tumor growth in prostate, liver, and breast cancers^53,54^. These results correspond with previous reports showing that melatonin depletion by light exposure late at night stimulates the growth of multiple human cancer xenografts and increases anticancer drug resistance^55,56^. Other experimental human studies with a simulated night shift work model have shown that bright light induces complete and rapid adjustment of peripheral clocks, suggesting that phototherapy may be a potential nonpharmacological intervention to counteract the progressive effects of shift work or jet lag on tumors^57,58^. Overall, these studies suggest that targeting the brain–neurohormone axis holds promise for achieving multiple therapeutic benefits in cancer prevention and treatment via the improvement or correction of circadian rhythms.

In recent studies, nutritional interventions have been increasingly thought to improve circadian rhythms and health^59^. Indeed, mouse studies have revealed that circadian molecular and metabolic profiles altered by aging are reverted by caloric restriction (CR), a well-known antiaging dietary intervention^60^. In addition to its antiaging benefits, increasing preclinical evidence indicates that CR may have anticancer effects by reducing tumor progression, enhancing the death of cancer cells, and elevating the effectiveness and tolerability of chemo- and radiotherapies^61^. Nonetheless, it is increasingly recognized that chronic CR often has detrimental effects on tumor development and chemotherapy in cancer patients, possibly by negatively affecting the immune system, wound healing, and other important functions^62^. As an alternative, intermittent fasting (IF), a diet-based therapy that alternates between fasting and free feeding/eating for a period of time, has been reported to inhibit tumor growth and improve antitumor immune responses in preclinical and clinical studies^63^. Furthermore, IF can increase cancer sensitivity to chemotherapy and radiotherapy and reduce the side effects of traditional anticancer treatments^63^. Taken together, this body of evidence suggests that well-designed chronodietary intervention holds promise as a potential therapeutic regimen to counter cancer, with fewer side effects and more safety.

Along with diet, exercise is gaining emerging attention as a potential chronotherapeutic intervention strategy for the prevention and treatment of multiple disease processes^17^. Notably, increasing numbers of human studies have revealed the therapeutic benefits of exercise in cancer treatment and survival^64^. Compared to mice treated with chemotherapy alone, mice treated with exercise plus chemotherapy presented with delayed tumor growth in models of breast cancer, melanoma, and pancreatic cancer^64^. However, most of these studies were performed without considering the effects of exercise timing on these disease processes. In this regard, a recent, population-based, case-control study reports that those who exercise in the early morning may have reduced risks of developing prostate or breast cancer than those who exercise in the evening^65^. In the future, it would be interesting to investigate how the timing of exercise influences cancer progression and chemotherapeutic outcomes with more mechanistic approaches.

In parallel with nonpharmacological interventions, several experimental and clinical studies have suggested that melatonin is an effective anticancer hormone beyond its circadian entrainment function^66^. A meta-analysis of randomized controlled trials revealed that low secretion of melatonin is associated with a higher incidence of cancer development in patients with exposure to light during nighttime hours^67^. Moreover, melatonin has the capacity to act specifically on cancer cells, not on normal cells^68^. Melatonin also reduces chemotherapy‐induced toxicity on normal cells through the re‐establishment of the light/dark circadian rhythm^69^. These results hold promise for establishing melatonin therapy as one of the safest chronobiotic strategies in the treatment of cancer.

Similar to melatonin, glucocorticoids (GCs) are also anticancer hormones. GCs are steroid hormones that are rhythmically secreted from the adrenal gland via SCN modulation of the hypothalamic–pituitary–adrenal stress response axis^70^. GC levels peak in the morning as a strong circadian cue to reset the peripheral tissue clock, and they have a systemic influence on metabolic (e.g., gluconeogenesis) and immune (e.g., anti-inflammatory) functions^70^. The synthetic GC steroid dexamethasone (Dex) has been used as a supportive care comedication for cancer patients undergoing standard care pemetrexed/platinum doublet chemotherapy^71^. In particular, GCs have been very effective in the treatment of lymphoid malignancies, including leukemia, lymphomas, and MM, with much work being done to enhance their effects and overcome resistance^72^. Interestingly, a recent animal study showed that enhancing the circadian clock with Dex treatment reduced tumor proliferation and growth in mouse melanoma^73^. Despite their tumor suppressor function, recent clinical reports suggest that GCs may also be implicated in poorer responses to cancer therapies with immune checkpoint inhibitors, such as anti-PD-(L)1, due to their immunosuppressive functions^74^. Given the diurnal fluctuation in endogenous GC levels, it would therefore be important to consider the dose and administration timing of corticosteroids to improve efficacy and safety in their use with immune-based cancer therapies.

#### Drugging clocks for cancer treatment

In recent years, the identification of a growing number of small molecules that modulate circadian rhythms by targeting core or noncore clock proteins has expanded the treatment windows and options for patients with diverse clock-related disorders^75^. Casein kinases 1δ and 1ε (CK1δ/ε) are critical components of the circadian clockwork that determine the circadian period and re-entrainment kinetics via phosphorylation of PERs to regulate their timed nuclear entry and activity^76^. Recently, casein kinases have been suggested to be pro-oncogenic proteins that are emerging as therapeutic targets in cancer^77^. For example, a series of potent and selective CK1δ/ε inhibitors have been shown to have an antitumor effect in the treatment of breast cancer both in vitro and in vivo^78^. Likewise, the CK1δ/ε inhibitor IC261 decreases cell survival and proliferation and increases apoptosis in colon, liver, and other human cancer cells, involving multiple oncogenic mechanisms^79^. In addition to studies targeting the CK1 family, more recent cell-based screens have identified a novel, potent, and highly selective CK2 inhibitor (GO289) that was demonstrated to strongly lengthen the circadian period and inhibit the growth of human and mouse cancer cells^80^. In line with these findings, several preclinical and clinical studies have shown that multiple anticancer drugs (e.g., umbralisib and CX-4945) targeting the CK1 or CK2 family exhibit unique immunomodulatory effects in the treatment of hematological cancers, such as chronic lymphocytic leukemia, other non-Hodgkin lymphomas, myelodysplastic syndrome, AML and multiple myeloma (MM)^81,82^. Collectively, these results suggest that targeting CK family members is a promising therapeutic avenue for the treatment of various forms of cancer.

Similarly, growing evidence suggests that pharmacological targeting of REV-ERBs may have therapeutic potential in a wide range of neuropsychiatric, metabolic, and immune disorders, as well as cancers. Synthetic REV-ERB agonists, such as SR9009 and SR9011, induce wakefulness, suppress sleep, regulate emotional behavior, and reduce anxiety-like behavior in mice^83^. Notably, recent in vitro and in vivo studies have revealed that REV-ERB agonists (SR9009 and SR9011) exhibit selective anticancer properties in brain, leukemia, breast, colon, and melanoma cancer cell lines by inhibiting autophagy and de novo lipogenesis important for cancer cell survival^45,84,85^. The antitumor effect of the REV-ERB agonist SR9009 was further confirmed by subsequent studies showing that treatment with this drug significantly reduced cell proliferation and viability in glioma cells as well as chemoresistant and chemosensitive small-cell lung carcinoma cells by impairing autophagy^86,87^.

Additional potential targets for treating cancer include several ROR isoforms and positive transcriptional regulators of *Bmal1* expression. Notably, emerging studies report that pharmacological inhibition of RORγ in vitro and in vivo exerts potent antitumor activity in multiple cancer types, such as castration-resistant prostate cancer^88^, pancreatic adenocarcinoma^89^, and triple-negative breast cancer^90^. On the other hand, synthetic RORγ agonists have also been reported to enhance antitumor immunity by increasing cytotoxic lymphocyte functions and attenuating immunosuppressive mechanisms^91^. Indeed, the ROR agonist LYC-55716 has been actively implemented in preclinical and clinical trials, showing good tolerability, safety, and pharmacokinetics in single or combined treatment with existing immunotherapeutic agents^74^. Notably, nobiletin, a dietary flavonoid found in citrus fruits, has been recently characterized as an agonist of RORα and RORγ that can induce apoptosis and cell cycle arrest, suppress migration and invasion, inhibit many oncogenic drivers, upregulate tumor suppressors, and increase chemotherapy sensitivity across several cancer cell types^92^. These results suggest that ROR isoforms may be a promising target for the treatment of cancers.

Increasing numbers of reports have suggested a potential role for CRY modulators in metabolic disease and cancer treatments. Hirota and colleagues first reported that KL001, a CRY stabilizer identified via cell-based high-throughput circadian assays, was found to lengthen the circadian period in a variety of cells and tissues and block glucagon-dependent induction of gluconeogenesis in cultured hepatocytes^93^. Remarkably, in a recent study, KL001 exhibited antitumor activity, such as impaired self-renewal, cell migration, and proliferation, as well as increased apoptosis, in patient-derived GBM stem cells^45^. However, another study showed that KL001 promoted cancer cell migration but had no effects on cell proliferation or colony formation in U2OS human osteosarcoma cells^94^. Interestingly, the CRY1/2 inhibitor KS15 has been reported to reduce MCF-7 cell growth and increase chemosensitivity in human breast cancer cells, possibly via the drug-induced elevation of PER2, a tumor suppressor clock protein^95^. These findings suggest that the antitumor effects of CRY modulators in cancer cells involve divergent mechanisms that are cell type-specific.

#### Clocking medicine for cancer treatment

**Fig. 5:**
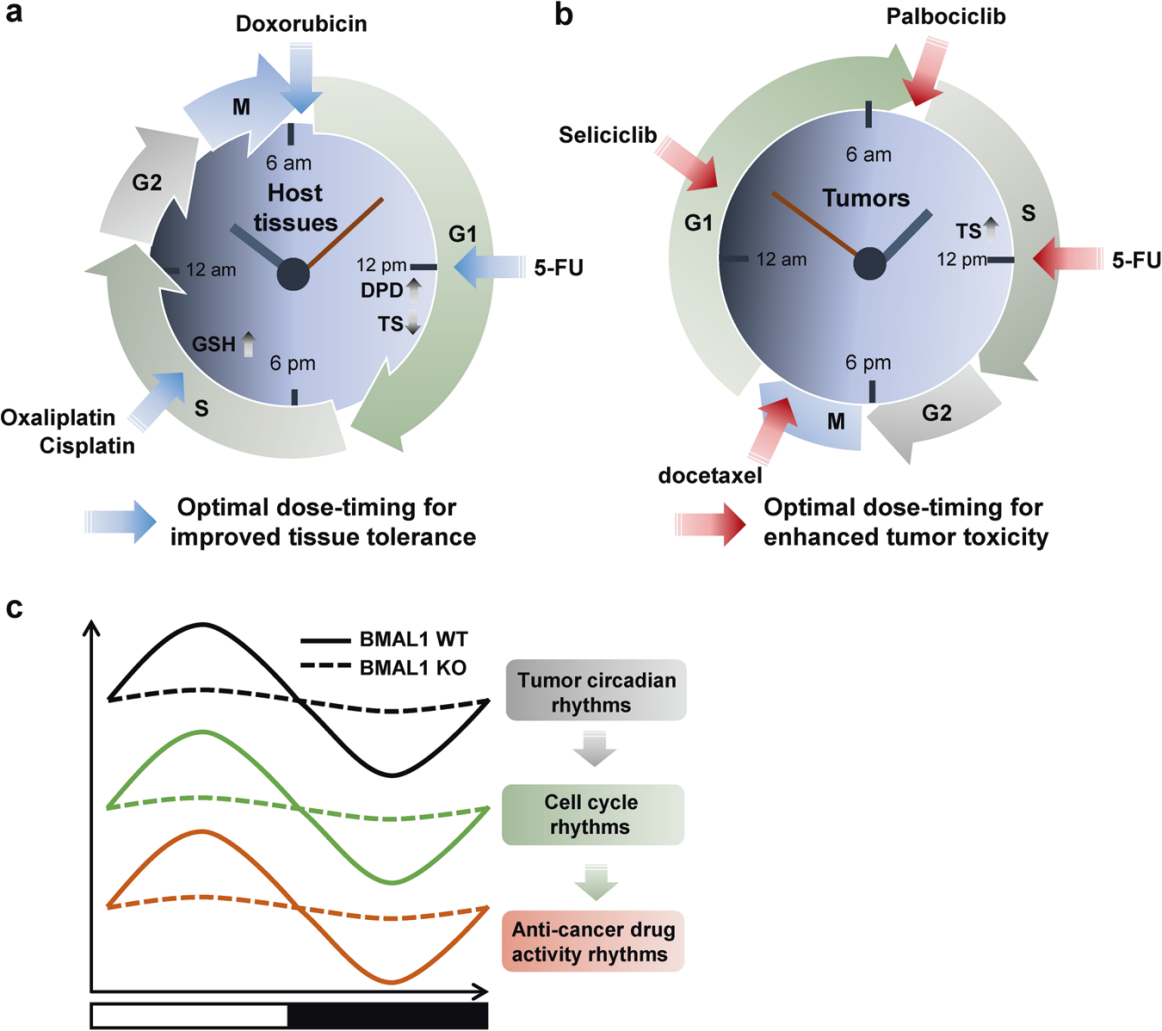
Circadian timing of cancer medicine. The purpose of chronotherapy in anticancer medicine is to improve host tolerance and safety (**a**) or tumor cytotoxicity (**b**). **a** Chronotissue tolerance in anticancer therapy. The antiphasic peak and trough levels of dihydropyrimidine dehydrogenase (DPD), an elimination enzyme of 5-FU, and thymidine synthase (TS) in host tissues are associated with reduced toxicity with 5-FU treatment. Daily variation in the levels of glutathione (GSH), a potent antioxidant, is another host chronotolerance biomarker to consider when platinum-based anticancer drugs (e.g., oxaliplatin and cisplatin) are used. For example, doxorubicin is more effective and causes fewer side effects with morning treatment. **b** Chronotumor toxicity of antitumor agents. Daily rhythms in the tumor-specific cell cycle can be targeted by various cell cycle-specific anticancer drugs, including those that target the G1 phase (seliciclib), G–S phase transition (palbociclib), S phase (5-FU), and M phase (docetaxel). G; cell growth, S; DNA synthesis, M; mitosis. **c** Circadian regulation of the time-of-day specificity of antitumor drugs. Circadian clock function in tumors regulates cell cycle rhythms that mediate the dose-time-dependent cytotoxicity of antitumor drugs. Genetic ablation of *BMAL1* (*BMAL1* KO; dashed line) in tumors abolishes cell cycle rhythms and the time-of-day-specific drug sensitivity present in intact tumor cells (*BMAL1* WT; solid line).

It has been well documented that circadian clocks regulate the absorption, distribution, metabolism, and elimination of drugs^96^. Furthermore, growing experimental and clinical evidence suggests that the circadian timing of medicine can be an important parameter in disease treatment, including chemotherapy^7^. Numerous clinical studies have shown that the efficacies of over 30 chemotherapy drugs can vary by over 50% depending on when they are administered^97^. Due to the cytotoxic effects of anticancer drugs on normal tissues as well as malignant cells, a major goal of chronocancer therapeutic studies is to reduce the toxicity that results in host tissue damage and immunological dysfunction (Fig. 5a). Early human studies in patients with advanced ovarian cancer showed that administration of doxorubicin in the morning (e.g., at 6 am) and cisplatin in the evening (e.g., at 6 pm before or after doxorubicin) caused fewer complications and less renal toxicity, along with dose reductions and treatment delays, when compared with administration of doxorubicin in the evening and cisplatin in the morning^98,99^. Likewise, in several clinical trials with patients with metastatic colorectal cancer, chronotherapy with irinotecan, oxaliplatin, 5-fluorouracil (5-FU), and leucovorin in combination resulted in advantages in time to progression and overall survival, with increased tolerability and safety compared to routine chemotherapy^100^.

Importantly, extensive studies have unraveled potential mechanisms underlying chronomodulated chemodrug safety. Computational analysis with experimental and clinical results has revealed that the same temporal pattern of drug (e.g., 5-FU and oxaliplatin) administration can result in minimal cytotoxicity in one cell population (e.g., normal cells), while at the same time, it can display high cytotoxicity in another cell population (e.g., tumor cells)^101^. These analyses underscore a potential advantage of chronotherapy in improving the simultaneous chronotolerance and chronoefficacy of anticancer drugs. For example, at the molecular level, the rhythmic response and toxicity of 5-FU are dependent upon circadian oscillations in thymidylate synthase activity, its molecular target, and dihydropyrimidine dehydrogenase activity (DPD), the rate-limiting enzyme responsible for its elimination^102,103^. In line with this, a recent clinical study has shown that circadian oscillation of the DPD enzyme, with the peak of activity occurring at 4 pm and the trough of activity occurring at 4 am, modulates the time-dependent bioavailability and efficacy of 5-FU^104^. Moreover, the levels of glutathione (GSH), an antioxidant tripeptide involved in drug detoxification, were also found to fluctuate daily, with peak concentrations occurring in the afternoon (~4 pm); therefore, platinum drug (e.g., cisplatin and oxaliplatin) toxicities were reduced when they were administered within the peak GSH time window^105^. Indeed, current knowledge of these circadian mechanisms has been widely leveraged to design and implement recent clinical trials, such as the induction of chronomodulated chemotherapy with 5-FU or cisplatin plus radiotherapy for NPC, with growing results showing reductions in adverse effects and enhanced tolerance with thoughtful, timed treatment approaches^106^.

In addition to host circadian rhythms, it has been suggested that daily cell-cycle dynamics in tumors can also be targeted for the time-of-day efficacy of anticancer therapy (Fig. 5b). In particular, cell cycle rhythm has been considered one of the most determinant factors in chronocancer therapy since 24-h mitotic rhythmicity was reported in human mammary cell cancer biopsy samples^107^. Notably, a flow cytometry study of cells from ovarian cancer patients revealed that tumor cell proliferation, as determined by the percentage of cells in the S (DNA synthesis) phase, exhibits a highly significant 24-hour rhythm, with a peak in the mid-to-late morning that is nearly 12 h out of phase with nontumor cell proliferation in normal tissues^108^ (Fig. 5a, b). Along these lines, preclinical and clinical cancer studies reporting on the chronoutility of cell cycle phase-specific drugs, such as cisplatin or 5-fluorouracil (5-FU) (S phase-specific), docetaxel (M phase-specific), and seliciclib (G1 phase-specific), showed that their maximal efficacy and minimal toxicity were achieved at different times during the day^109^. Furthermore, palbociclib (PD-0332991), a selective inhibitor of cyclin-dependent kinase (CDK) 4/6, which is responsible for G1/S cell cycle progression, was found to reduce the growth of cultured cells and mouse tumors in a time-of-day-specific manner^3^. Together, these findings argue for chemotherapy approaches that are timed to coincide with times of high tumor cell vulnerability and low toxicity to normal tissues^108^ (Fig. 5).

Increasing evidence suggests direct roles for circadian clock genes in modulating the efficacy of anticancer therapies depending on the time of day. In recent individual chronopharmacological studies, the DNA alkylator temozolomide and irinotecan, a topoisomerase I inhibitor, was shown to exhibit rhythmic drug toxicity in GBM and colorectal cancer cells, respectively, with maximum drug sensitivity occurring near the peak of *BMAL1* expression. These cytotoxic rhythms were ablated when *BMAL1* was silenced^110,111^. These studies coincide with other reports indicating that elevated *BMAL1* expression increases the sensitivity of colorectal cancer and TSCC cells to oxaliplatin and paclitaxel^28,112^. Similar to BMAL1, a recent mouse tumor graft study with human oral squamous cell carcinoma (OSCC) cells suggested that PER2 is an effective chronomodulator of DNA damaging agents (e.g., oxaliplatin) since the efficacies of these drugs can be greatly boosted with timely administration at the peak of PER2 expression^113^. In further molecular analyses, PER2 was shown to periodically suppress proliferating cell nuclear antigen transcription, which, in turn, impeded the drug-induced DNA repair mechanism, thus increasing chronodrug sensitivity^113^. In addition, other individual studies using in vitro or in vivo tumor graft models showed that synthetic (e.g., SR9009, bortezomib, and aldehyde dehydrogenase inhibitor) and natural (e.g., curcumin) compounds could exert time-of-day-dependent antitumor activity via multiple circadian-dependent metabolic factors in GBM, liver, and breast cancer cells^86,114,115^. In line with these findings, a recent large-scale chronopharmacological analysis revealed that multiple anticancer drugs, including HSP90 inhibitors, exhibit time-of-day cytotoxicity, which requires cell cycle rhythms; however, these effects were abrogated in clock-deficient cancer cells^116^. In further analyses, *Bmal1* ablation was shown to critically affect melanoma proliferation and time-of-day sensitivity to an HSP90 inhibitor in vivo^116^. Overall, these results clearly suggest that circadian regulation of cellular dynamics in tumors can dictate the time-of-day specificity of anticancer drugs (Fig. 5c).

Notably, it is increasingly recognized that chronotype, sex, age, disease status, and other interpersonal differences in rhythm status can largely affect pharmacodynamic variability among cancer patients, posing a daunting challenge in cancer drug therapy^2^. In particular, growing numbers of experimental and clinical studies suggest that environmental or physiological perturbations of circadian rhythms, such as shift work, abnormal sleep timing, irregular psychosociological stresses, and critical illness, can underlie interindividual variability in both cancer growth and response to cancer therapy^3,117^. Circadian disruption may also be related to the chronic sleep loss and depression suffered by many cancer patients following diagnosis and treatment^118^. Thus, it can be anticipated that training or enhancing the body clock with scheduled light exposure, mealtimes, or exercise, alongside a carefully timed chemotherapy regimen, would improve antitumor treatment.

## Concluding remarks

In recent decades, extensive chronobiological research has expanded our understanding of the functional roles and mechanisms of the circadian clockwork in human health and diseases, including cancer. Circadian disruptions negatively influence both tumor molecular clocks and host circadian systems to increase cancer risk and progression. On the other hand, given the close link between cancers and other rhythm-disruptive pathologies, such as aging, obesity, and diabetes, enhancing circadian rhythms with chronophysiological interventions (e.g., chronophototherapy, chronodiet, and chronoexercise) is expected to highly benefit overall circadian health and cancer therapy. In addition, the dose timing of chemotherapeutic agents is an important therapeutic option that can improve tumor-specific cytotoxicity while sparing normal cells and host tissues. With the growing development of chronotherapeutic tools and strategies, it may be reasonable to expect that integrative chronotherapy will further improve the prevention and treatment of cancer.
